# Prioritizing Cancer Genes Based on an Improved Random Walk Method

**DOI:** 10.3389/fgene.2020.00377

**Published:** 2020-04-28

**Authors:** Pi-Jing Wei, Fang-Xiang Wu, Junfeng Xia, Yansen Su, Jing Wang, Chun-Hou Zheng

**Affiliations:** ^1^Key Lab of Intelligent Computing and Signal Processing of Ministry of Education, College of Computer Science and Technology, Anhui University, Hefei, China; ^2^Division of Biomedical Engineering, University of Saskatchewan, Saskatoon, SK, Canada; ^3^Department of Computer Sciences, University of Saskatchewan, Saskatoon, SK, Canada; ^4^Department of Mechanical Engineering, University of Saskatchewan, Saskatoon, SK, Canada; ^5^Institutes of Physical Science and Information Technology, Anhui University, Hefei, China; ^6^College of Computer and Information Engineering, Fuyang Normal University, Fuyang, China

**Keywords:** cancer, driver gene, protein–protein network, random walk, centrality

## Abstract

Identifying driver genes that contribute to cancer progression from numerous passenger genes, although a central goal, is a major challenge. The protein–protein interaction network provides convenient and reasonable assistance for driver gene discovery. Random walk–based methods have been widely used to prioritize nodes in social or biological networks. However, most studies select the next arriving node uniformly from the random walker's neighbors. Few consider transiting preference according to the degree of random walker's neighbors. In this study, based on the random walk method, we propose a novel approach named Driver_IRW (Driver genes discovery with Improved Random Walk method), to prioritize cancer genes in cancer-related network. The key idea of Driver_IRW is to assign different transition probabilities for different edges of a constructed cancer-related network in accordance with the degree of the nodes' neighbors. Furthermore, the global centrality (here is betweenness centrality) and Katz feedback centrality are incorporated into the framework to evaluate the probability to walk to the seed nodes. Experimental results on four cancer types indicate that Driver_IRW performs more efficiently than some previously published methods for uncovering known cancer-related genes. In conclusion, our method can aid in prioritizing cancer-related genes and complement traditional frequency and network-based methods.

## Introduction

As one of the most complex and threatening diseases, cancer has attracted the attention of many research groups and large-scale programs [such as The Cancer Genome Atlas (TCGA) (Network, 2008) and the International Cancer Genome Consortium (Bobrow and Zhao, 2010)] to explore the molecular mechanisms and pathogenesis. With the rapid advances of technology, huge volumes of cancer genomics data have been generated containing many different types of genetic aberrations, such as single-nucleotide variants (SNVs), copy number variations (CNVs), and small and large insertions and deletions (Indels) (Zhang and Zhang, 2017; Dimitrakopoulos et al., 2018). It has been demonstrated that cancer is related to gene mutations, but only a few genes exist that confer selective growth advantage to cancer progression, known as driver genes. The remaining mutations are called passenger genes (Greenman et al., 2007; Stratton et al., 2009; Vogelstein et al., 2013; Iranzo et al., 2018). However, identifying and distinguishing driver genes from myriad passengers are a fundamental question and an intractable challenge (Haber and Settleman, 2007; Stratton et al., 2009; Vogelstein et al., 2013) and are crucial to gain insights into biological processes (Zhang et al., 2018).

Efforts have been made to address this challenge using a variety of novel methods. The most traditional approaches are based on genetic aberration frequencies among population cohorts of patients with cancer (Gui et al., 2011; Dees et al., 2012; Lawrence et al., 2013), which can detect some major driver genes with significantly higher mutation rates than background mutation rates (BMRs). However, the estimations of BMR significantly affect the identification of driver mutations. Additionally, it has been found that the BMR is dependent on the genomic context of the nucleotide, the type of mutation transcription rates, and replication time (Raphael et al., 2014). Therefore, it is difficult to estimate BMR accurately. Moreover, genes altered in only a few individuals may be relatively important in contributing to cancer progression (Stratton et al., 2009; Raphael et al., 2014; Hristov and Singh, 2017). Evidently, these frequency-based methods cannot reveal rarely mutated driver genes. Consequently, some promising methods considering somatic mutations in the context of pathways are proposed, because cellular signaling and regulatory pathways are usually affected by driver mutations (Network, 2008; Vandin et al., 2012; Jones et al., 2016). Additionally, most pathway-based methods are primarily based on the mutual exclusivity of mutations (Zhang et al., 2014; Wu et al., 2015; Zhang and Zhang, 2018). They are focused on analyzing somatic mutation rather than integrating different omics data, such as transcriptome and interactome. Given that proteins tend to be proximal if they take part in the same pathways (Hristov and Singh, 2017), in recent years, many novel methods based on networks have been successfully applied to cancer driver gene identification by integrating different omics data (Bashashati et al., 2012; Hou and Ma, 2014; Amgalan and Lee, 2015; Bertrand et al., 2015; Dimitrakopoulos et al., 2018; Song et al., 2019). However, some only map genes of different omics data into networks without collecting more information regarding network topology into account. Furthermore, some network diffusion approaches, such as DawnRank (Hou and Ma, 2014), propagate expression information through a protein interaction network by selecting the next arriving node from its neighbors uniformly. Moreover, Gentili et al. (2019) have proposed a BRW (biological random walk) method to leverage biological information in network propagation for gene prioritization. In addition, there are also some advances of random walk in different research field. For example, Chen et al. (2016) have proposed an improved random walk with restart method for lncRNA-disease association prediction (IRWRLDA). These two methods improve the initial probabilities of restart term by setting uniform probability of disease-associated seed nodes and considering lncRNA expression similarity and disease semantic similarity separately. However, in real-world scenarios, the random walker is more likely to have tendentiousness and preference for selecting the neighbors with a greater degree rather than uniformly (Liu et al., 2017). The aforementioned random walk–based methods rarely consider it. Although some methods have realized the importance of seed genes, they did not consider the topological characteristics. Therefore, it is beneficial for a novel method to take more graph topological characteristics and propagating tendency into consideration to identify cancer driver genes.

In this study, to mitigate these methodological limitations and improve the accuracy of driver gene identification, we proposed a novel approach based on the random walk method, named Driver_IRW (Driver genes discovery with Improved Random Walk method), for driver genes discovery by integrating transcriptomic data and interaction network. The assumption of our method is that genes in the interaction network with a higher degree have a higher transition probability from their upstream neighbors. First, we constructed different networks for different types of cancer by selecting those edges that exist in both the known PPI network and differential coexpression network (Guo et al., 2019), in which the known information of the PPI network used is a directed network from DanwRank (Hou and Ma, 2014). The tumor and normal expression data were used to construct the differential coexpression network for each type of cancer. Then, the degree, betweenness, and Katz centralities were obtained based on the constructed network. Third, based on the assumption, we adopt the strategy that the information in network is diffused in accordance with its neighbors' out-degree rather than uniformly (Liu et al., 2017). Moreover, the betweenness and Katz centrality of different seed genes of corresponding cancers were merged as random jumping probabilities to these nodes, in which the different seed nodes were extracted from CGC (Sondka et al., 2018) and DisGeNet databases (Piñero et al., 2016) for different cancers. The random walk scores were calculated by the improved random walk method. Finally, only mutated genes were retained. To evaluate the performance of the proposed method, data of four cancer types from TCGA were used, and the results indicate that it performs well. Moreover, the benchmark analysis showed that the proposed method is useful.

## Materials and Methods

### Datasets

In this work, four different types of cancer, breast cancer (BRCA) with 1097 samples, head and neck squamous cell carcinoma (HNSC) with 522 samples, kidney renal cell cancer (KIRC) with 534 samples, and thyroid cancer (THCA) with 513 samples, from TCGA were studied. The datasets used consisted of mutation and expression data from tumor and normal samples for every cancer. The mutation data were integrated by SNVs and CNVs. It was regarded as a mutated gene if there was an SNV or CNV present, in which the CNV data are downloaded from UCSC data portal (http://xena.ucsc.edu/) (Rosenbloom et al., 2015), which have transformed the data from TCGA using Gistic2 method. And we retained only those genes with +2 and −2 values, which are the high-level amplification and homozygous deletion. The RNAseq expression data were real values denoting the normalized abundance of each gene in each sample. To obtain the differential coexpression network, the expression data of normal and tumor samples were required. In addition, the seed genes of different cancers were derived from CGC (release v85, downloaded on May 8, 2018) (Sondka et al., 2018) and DisGeNet (release v6, downloaded in February, 2019) databases (Piñero et al., 2016). The CGC database encompasses 719 expert-curated descriptions of the genes driving human cancer (Sondka et al., 2018), and DisGeNet is one of the largest available collections of human disease-involved genes and variants (Piñero et al., 2016). Additionally, the reference network used in this study was downloaded from DanwRank (Hou and Ma, 2014), which integrates various sources, including MEMo (Ciriello et al., 2012), NCI-Nature Curated PID (Schaefer et al., 2008), Rectome (Croft et al., 2010), and KEGG (Kanehisa et al., 2011). It can be viewed as a directed graph.

### The Construction of the Cancer-Related Network

To retrieve more specific peculiarity of different types of cancer, we constructed different networks for different types of cancer by integrating the known PPI network and differential coexpression network (Guo et al., 2019).

**Figure 1:**
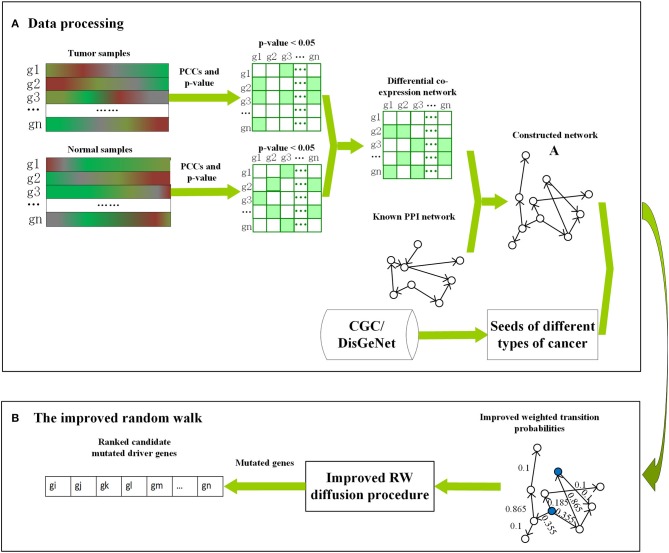
The framework of Driver_IRW for identifying cancer driver genes. **(A)** The procedure of data processing, including the construction of the network and the selection of the seed genes for different cancers. **(B)** The improved random walk, including the calculation of the improved transition probabilities and the calculation of the global centralities for seed genes (blue nodes). The mutation data are used as posterior information to filter the mutated candidate driver genes.

First, Pearson correlation coefficients with *p*-values of tumor and normal expression data were calculated separately as coexpression networks for different cancers. Then, the differential coexpression network was constructed as per the following two steps: (1) only the edges with *p* < 0.05 were selected and assigned to 1 as significantly correlated gene pairs; (2) the differential edges that were significantly correlated only in the tumor or normal coexpression networks, i.e., the edges, were statistically significant (*p* < 0.05) in tumor (normal) data but not (*p-value* > 0.05) in normal (tumor) data, were screened out. The consistent edges, i.e., edges that were statistically significant or not in both tumor and normal coexpression networks, were removed. Finally, the reference network downloaded from DawnRank (Hou and Ma, 2014) was integrated with differential coexpression network by selecting the common nodes and edges (Guo et al., 2019). The framework of Driver_IRW is shown in Figure 1. This reconstructed network, i.e., adjacency matrix, is a 0–1 matrix represented as *A* with *A*(*i, j*) = 1 if node *i* links to *j* in the constructed network, otherwise, *A*(*i, j*) = 0.

### The Selection of Cancer-Related Seed Genes

In previous studies, seed genes have been widely used as prior information for disease gene discovery (Köhler et al., 2008; Moreau and Tranchevent, 2012). To take this prior information into account in the Driver_IRW framework, and in light of the previous conception, known cancer-related genes for the corresponding types of cancer were used as seed genes in this study. Different cancer-related genes were extracted for corresponding types of cancers from CGC (release v85, May 8th, 2018) (Sondka et al., 2018) and DisGeNet (Piñero et al., 2016) databases.

### The Calculation of Centralities

By virtue of the adjacency matrix, the topological centralities, here are degree centrality, betweenness centrality, and Katz feedback centrality (termed as *DC, BC*, and *KC*, respectively), were calculated. The degree centrality was used to obtain the weighted transition matrix. Additionally, the betweenness and Katz feedback centralities were used to evaluate the random jumping probability to seed genes.

The degree centrality of a vertex is the number of edges incident to the vertex in a graph. That is,

(1) $$\begin{array}{r} DC_{i}=\sum_{j=1}^{n}A_{ij} \end{array}$$

where *n* is the total number of genes in the network; *A*_*ij*_ is the adjacency matrix of the network.

The betweenness centrality can be interpreted as a vertex being more central if it is needed to transport more information of others in the network. This is calculated as follows:

(2) $$\begin{array}{r} BC_{i}=\frac{2}{n^{2}-3n+2}\sum_{s\neq i\neq t}\frac{n_{st}\left(i\right)}{g_{st}} \end{array}$$

where *g*_*st*_ indicates the total number of shortest paths from node *s* to node *t*, and *n*_*st*_(*i*) indicates the number of these paths that pass through vertex *i*. The betweenness centrality was obtained using the “*igraph*” R package, which provided handy tools to create, manipulate, and visualize networks, and calculate various structural properties (Csardi, 2006).

The Katz centrality, one of the feedback centralities, is calculated based on the impact of a vertex on others. This is defined as follows:

(3) $$\begin{array}{r} KC\left(i\right)=\sum_{k=0}^{\infty }\sum_{j=1}^{n}\alpha^{k}{\left(A^{k}\right)}_{ji} \end{array}$$

where *A* is the adjacency matrix; ${\left(A^{k}\right)}_{ji}$ is the number of paths from *j* to *i* with length *k*; and α is a damping factor, which restricts that the longer the path between *i* and *j*, the smaller the impact of *i* on *j* should be. It has been proved that to guarantee convergence the α must be restricted as follows:

(4) $$\begin{array}{r} \lambda_{1}<\frac{1}{\alpha }\Leftrightarrow \sum_{k=1}^{\infty }\;\alpha^{k}A^{k}\;converge \end{array}$$

where λ_1_ is the largest eigenvalue of *A*. The closed form expression is Equation (5) when it converges:

(5) $$\begin{array}{r} KC=\sum_{k=1}^{\infty }\alpha^{k}{\left(A^{T}\right)}^{k}1_{n}=\left({\left(I-\alpha A^{T}\right)}^{-1}\right)1_{n} \end{array}$$

Then, the prior information used in the diffusing procedure as random jumping probabilities to seeds is represented by the normalized mean value of normalized betweenness centrality and Katz centrality of seeds.

### The Algorithm of Improved RW

In light of the above assumption that the information in the network is diffused in accordance with its neighbors' out-degree instead of uniformly, the transition matrix whose values represent transition probability from the vertex *i* to any vertex *j* of the directed graph was defined as follows (Liu et al., 2017):

(6) $$\begin{array}{r} p_{ij}=\{\begin{array}{c} \alpha \frac{DC_{j}^{+}A_{ij}}{\sum_{v\in N^{+}\left(i\right)}DC_{v}^{+}}+\left(1-\alpha \right)\frac{1}{n},if\;\sum_{v\in N^{+}\left(i\right)}DC_{v}^{+}\neq 0;\\ \;\frac{1}{n},\;otherwise \end{array} \end{array}$$

where $DC_{v}^{+}$ and $DC_{j}^{+}$ are the out-degree of vertex *v* and *j*, respectively. *N*^+^(*i*) denotes all the neighbors interacting with vertex *i* in the network. In addition, *n* is the total number of genes in the network. This means that if there are neighbors of one node, the transition probabilities from the node to the neighbors are proportional to the neighbors' out-degree; otherwise, the transition probability is uniform according to the total genes. A parameter of α (empirically, here α = 0.85) is presented. This is used to avoid neglecting the nodes whose out-degree of the neighbors of node *i* is zero.

Next, we defined the score of each gene iteratively according to the improved random walk approach:

(7) $$\begin{array}{r} r_{i}\left(t+1\right)=d\cdot \sum_{j=1}^{n}p_{ji}\cdot r_{j}\left(t\right)+\left(1-d\right)prior\text{\_}p_{s} \end{array}$$

It can be presented in the matrix form:

(8) $$\begin{array}{r} r\left(t+1\right)=dP^{T}\times r\left(t\right)+\left(1-d\right)\times prior\text{\_}p \end{array}$$

where *r*(*t*) and *prior_p* are *n* × 1 vectors representing the gene score in the *t*-th iteration of each node and prior information of seed nodes separately with the sum of the values equal to 1 (Köhler et al., 2008). The initial gene scores *r*(0) are the normalized mean of all tumor expression. Moreover, *P*^*T*^ is the transposition of the transition matrix *P* obtained by Equation (6). Here, *d* is set to 0.85 according to the initial PageRank algorithm and is the damping factor that corresponds to a random walker periodically jumping to a random node (Page et al., 1999), which is used as the seed gene of different cancers. To retrieve more global information on the network, betweenness and Katz feedback centralities were used to represent the random jumping probabilities of a random walker jumping to the seeds in each iteration.

The final random walk scores of all nodes converged to a stationary distribution when there was no longer a significant update in the scores (Hou and Ma, 2014). When the difference (Equation 9) in scores between the (*t*+1)-th and previous *t*-th iteration was smaller than ε, the iteration stopped. Here, the threshold was set as ε = 1*e* − 8. Additionally, the iteration stopped after the maximum number of iterations, which was set to 1,000, when no solution was obtained. In practice, the improved method always converges to the stationary status.

(9) $$\begin{array}{r} diff=\sqrt{\sum_{i=1}^{n}{\left(r_{i}\left(t+1\right)-r_{i}\left(t\right)\right)}^{2}} \end{array}$$

To retrieve the impact of the mutated genes in the population, only the mutated genes were retained in the final results.

## Results

### Performance Evaluation for Known Cancer-Related Genes

**Figure 2:**
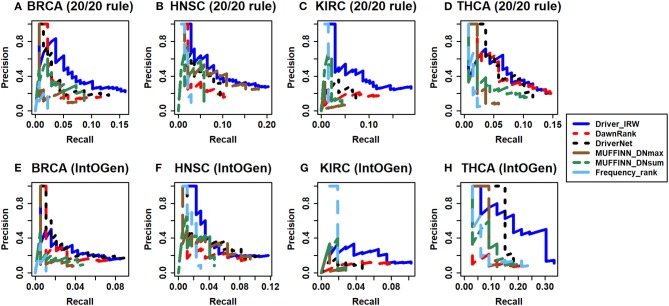
Assessment of predictive power of Driver-IRW for known cancer related genes compared with different methods. The precision-recall curves of the top 100 results of Driver-IRW, DawnRank, DriverNet, MUFFINN (contained two results of MUFFINN_DNmax and MUFFINN_DNsum), and naïve frequency-based methods by 20/20 rule gene list for four datasets **(A)** BRCA, **(B)** HNSC, **(C)** KIRC, and **(D)** THCA, and by IntOGen database for datasets **(E)** BRCA, **(F)** HNSC, **(G)** KIRC, and **(H)** THCA.

For evaluating and comparing the performance of Driver_IRW in predicting known cancer genes, two publicly available databases-20/20 rule (Vogelstein et al., 2013) and IntOGen (downloaded on May, 2019) (Gonzalez-Perez et al., 2013), datasets were utilized as approximate benchmarks. The 20/20 rule dataset contained 138 well-studied oncogenes and tumor suppressor genes, which were used to assess the ability of our method in identifying known cancer drivers. The IntOGen database lists previously detected drivers of different cancers, which were used to evaluate the performance of driver discovery on specific types of cancer. Different driver genes were extracted for different types of cancer as benchmarks for performance comparison. Therefore, we compared Driver_IRW with previous state-of-the-art methods, such as DawnRank (Hou and Ma, 2014), DriverNet (Bashashati et al., 2012), MUFFINN (Cho et al., 2016), and naive mutation frequency-based method to evaluate the performance of predicting known cancer-related genes. In particular, the MUFFINN method provided two strategies; DNmax (direct neighbor max), which counts mutations in the most frequently mutated neighbors, and DNsum (direct neighbor sum), which counts mutations in all direct neighbors using the networks HumanNet (Lee et al., 2011) and STRING (Szklarczyk et al., 2015; Cho et al., 2016). For comparison, the best performance between the two networks of each strategy of the top *N* genes was selected. In addition, other methods were executed in their default settings. Moreover, the precision-recall curves of the top *N* genes are illustrated in Figure 2. The precision and recall matrices are defined as follows:

(10) $$\begin{array}{r} \text{precision}=\frac{\left(\#\text{genes in benchmark})\cap (\#\text{ genes found in Driver}\_\text{IRW}\right)}{\#\text{genes found in Driver}\_\text{IRW}} \end{array}$$

(11) $$\begin{array}{r} \text{recall}=\frac{\left(\#\text{genes in benchmark})\cap (\#\text{ genes found in Driver}\_\text{IRW}\right)}{\#\text{genes found in benchmark}} \end{array}$$

where (# genes *in benchmark*) represents the number of genes in benchmarks (20/20 rule and IntOGen datasets) dataset, and (# genes *found in Drive r*_*IRW*_) represents the number of top N genes prioritized by Driver-IRW.

In practice, researchers may only be interested in the top-ranked candidate genes for follow-up experimental validation. Hence, only the top 100 candidate driver genes (detailed lists are in the Supplementary File) were selected to assess their performance. In general, Driver_IRW outperforms most other methods in four datasets regardless of whether assessing with the 20/20 rule or IntOGen metrics, which indicates that Driver_IRW can identify more known cancer drivers than other methods. Overall, the results show that our proposed method performs well in identifying known cancer-related genes.

### Analysis of the Improvement of the Transition Matrix

**Figure 3:**
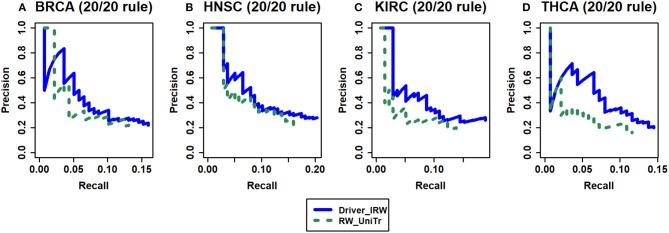
The analysis of the improvement of transition matrix. Driver_IRW is our proposed method; RW_UniTr means random walk method with uniform transition probability. The precision-recall curves of top 100 results of Driver_IRW and RW_UniTr by 20/20 rule gene list for four datasets **(A)** BRCA, **(B)** HNSC, **(C)** KIRC, and **(D)** THCA.

To verify that there are improvements after incorporating the tendency of nodes in random walk, the strategy that assigns all neighbors of node *i* with uniform transition probabilities (named RW_UniTr) was adopted. Here, the random jumping probability remained the same as that of Driver_IRW. The benchmark data are the 20/20 rule, and the results are shown in Figure 3. The results indicated that the transition tendency in accordance with the neighbors' out-degree and network topological information are important for improving the performance of the method.

### The Analysis of Seed Genes

**Figure 4:**
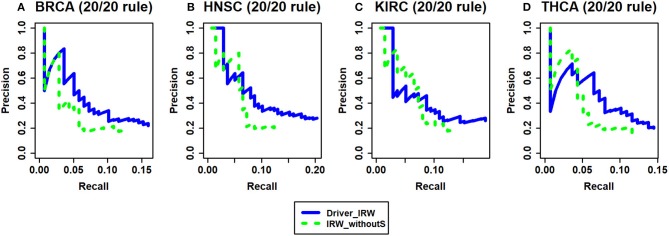
The analysis of seed genes. Driver_IRW is our proposed method with seed genes; IRW_withoutS means removing the seed genes from Driver_IRW method and setting them with equal probabilities. The precision-recall curves of top 100 results of Driver_IRW and IRW_withoutS by 20/20 rule gene list for four datasets **(A)** BRCA, **(B)** HNSC, **(C)** KIRC, and **(D)** THCA.

Based on the successful application of seed genes on disease-related discovery in previous studies (Köhler et al., 2008), known cancer driver genes were used in our method. To evaluate the performance after considering seed genes, the seed genes were removed from our Driver_IRW method (termed as IRW_withoutS). Without the seed genes, the random jumping probabilities of all nodes were set to equal values (1/*n, n* is the total number of genes), which was equivalent to letting the random walker jump to all nodes with equal probability. Analogous to the analysis of transition matrix, the benchmark here is the 20/20 rule dataset. The results are shown in Figure 4. These results indicate that there is mild improvement after taking the seed genes into consideration.

**Figure 5:**
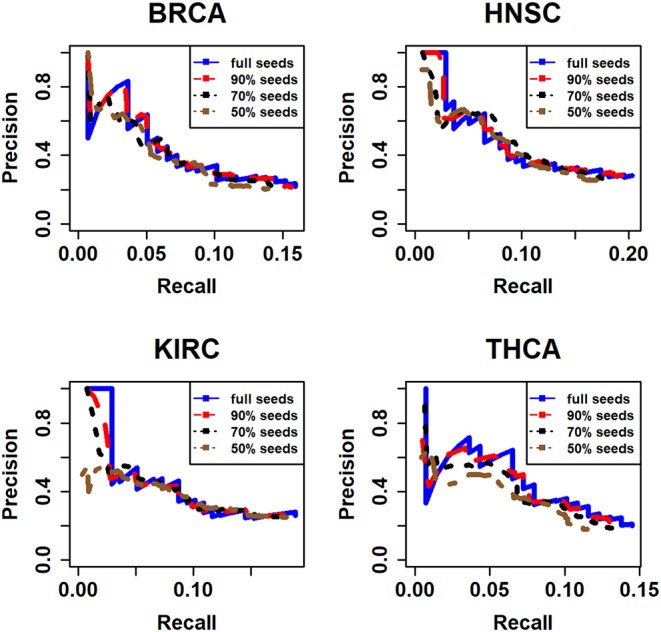
The assessment of different seeds for four types of cancer. Here, the full seeds mean the results of all seed nodes we integrated from CGC and DisGeNet database; 90% seeds, 70% seeds, and 50% seeds mean that we randomly deleted 10, 30, and 50% seed nodes, respectively.

In addition, in order to investigate the impact of the seed nodes and demonstrating whether the results are sensitive to the selection of seed node, we have randomly deleted 10, 30, and 50% nodes for 10 times, respectively, from all seed nodes we used in this article. Next, we applied our method to every subset. Then the accuracy and recall are calculated in accordance with the 20/20 rule dataset for 10 times. Finally, the mean value of accuracy and recall of 10 times are calculated and used to compared with the full seed nodes (Figure 5).

It is obviously that the performances of full seeds and 90% seeds are similar. And with the rise of the percentage we deleted seeds from full seeds, the performance seems becoming a little worse especially in KIRC and THCA dataset. However, in general, the performance on these subsets does not change significantly. It means that the results are not too sensitive to the selection of seed nodes.

### Analysis of the Top 10 Candidate Drivers

The overall performance of identifying cancer drivers of the top 100 genes is shown in Figure 2. Here, the top 10 candidate genes were listed to illustrate their importance. First, their statuses were explored regarding whether they are known cancer drivers or candidate drivers in the NCG 6.0 database (Repana et al., 2019), which is a manually curated repository that contained 2,372 genes whose somatic modifications have been reported as known or predicted cancer driver roles (Repana et al., 2019). Then, the mutation frequencies of these genes were calculated to investigate the sensibility to the mutation frequency of our method. The results are shown in Table 1.

**Table 1:** The top 10 candidate drivers of different cancers.

**BRCA**			**HNSC**		
**Candidate genes**	**Status in NCG6.0**	**No. of mutated**	**Candidate genes**	**Status in NCG6.0**	**No. of mutated**
*TP53*	Known	310	*TP53*	Known	362
*JUN*	Known	24	*AKT1*	Known	14
*CTNNB1*	Known	7	*EGFR*	Known	69
*EGFR*	Known	30	*PIK3CA*	Known	179
*AR*	Known	18	*ERBB3*	Known	17
*PIK3R1*	Known	29	*MAPK1*	Known	18
*SRC*	Known	25	*CTNNB1*	Known	10
*RELA*	Predicted	26	*MAPK3*	Unknown	1
*PAK1*	Unknown	94	*STAT3*	Known	8
*MYC*	Known	230	*PIK3R1*	Known	10
**KIRC**			**THCA**		
**Candidate genes**	**Status in NCG6.0**	**No. of mutated**	**Candidate genes**	**Status in NCG6.0**	**No. of mutated**
*CTNNB1*	Known	58	*HRAS*	Known	18
*TP53*	Known	6	*EIF1AX*	Known	9
*PIK3R1*	Known	1	*SRC*	Known	2
*PIK3CA*	Known	1	*TP53*	Known	4
*RELA*	Predicted	17	*NRAS*	Known	40
*MAX*	Known	5	*AKT1*	Known	6
*PAK1*	Unknown	3	*KRAS*	Known	5
*SRC*	Known	1	*TRIM24*	Known	1
*HIF1A*	Known	6	*CTNNB1*	Known	1
*EGFR*	Known	4	*HSP90AA1*	Known	3

As shown in Table 1, almost all of the top 10 genes are cancer-related according to NCG 6.0. Genes not reported by NCG 6.0 may also have potential effects on cancer. For example, it has been demonstrated that *PAK1* is increased in breast cancer and plays a pivotal role in promoting tumor growth and drug resistance (Kumar et al., 2006; Dou et al., 2016). In addition, *PAK1* has been reported by some studies to play a key role in the initiation and progression of KIRC (O'Sullivan et al., 2007).

Furthermore, besides the frequently mutated and important candidate drivers, such as *TP53* in BRCA and HNSC and *NRAS* and *HRAS* in THCA, rare mutated (usually defined as mutated frequency <2% of samples Hou and Ma, 2014) candidate drivers could also be identified through our method (Table 1). Interestingly, *TP53* and *CTNNB1* rank in the top 10 in four cancer types. It is widely known that the tumor suppressor gene *TP53* is frequently mutated in most human cancers and has an important role in the cellular stress response (Petitjean et al., 2007; Hidalgo, 2010). Additionally, *CTNNB1* is reported as a potential biomarker using the KIRC corresponding network (Isik and Ercan, 2017). Moreover, mutations in *CTNNB1* are related to several human malignancies, such as colorectal cancer (Klaus and Birchmeier, 2008), lung cancer (Schou et al., 2001), HNSC (Jerhammar et al., 2010), and KIRC (Hirata et al., 2012), although it is a rarely mutated gene.

## Discussion

The identification of cancer driver genes is a valuable task for cancer genomics analysis (Guo et al., 2018). In this study, we propose Driver_IRW, an improved random walk–based framework, to prioritize cancer genes. Since the transition of a node moving to others is more likely to have tendentiousness in reality, a strategy different from the traditional random walk method was used whereby nodes transit to others based on the out-degree of their neighbors rather than move to their neighbors uniformly according to the degree of themselves. This not only spreads information rapidly but also avoids trapping by dangling nodes. Additionally, the application of the method without seed genes exhibits that the seed genes play a role in driver gene identification. However, the improvement is not prominent. On the one hand, the quality of the seeds may affect the performance; hence, it is necessary to collect high-quality seeds. On the other hand, this might mean that our method is robust to the prior information. In a nutshell, the main differences of our proposed method Driver_IRW with other methods are mainly in two aspects. The first one is the computation of the transition probability compared with some methods such as DawnRank, BRW, and IRWRLDA. The second one is the computation of the prior information compared with DPRank method. DPRank method considers the tendentiousness of the neighbors' degree in the network; however, it neglects the impact of known seeds in the real biology network. The experimental results on four different cancer datasets (Figure 2) indicate that our method is more effective when evaluating known driver discovery than some previous methods. Moreover, the results in Table 1 also show that Driver_IRW can identify not only the frequently mutated genes but also rarely mutated drivers. Comparison of Driver_IRW with the method that uses traditional transition probability indicates that the improvement on transition matrix indeed improves the performance. It should be noted that Driver_IRW was only applied on four TCGA datasets as case studies in this study; it could be extended to other datasets if expression data, mutation data, and corresponding seeds genes are available.

Our method may be a complement of the traditional frequency-based methods and some network-based methods. However, there are also some limitations for this method. In this study, the mutation data are only regarded as the posterior information to filter the candidate driver genes. Generally, cancer evolves to accumulate additional alterations (Nussinov et al., 2019), which might infer more important information to take the mutation and time-dependent alteration data into consideration. Besides genetic aberrations, other events, such as miRNA differential expression and epigenetic changes, can also contribute to the progression of cancer. The expression of mRNAs can be controlled by upregulated miRNAs (Dimitrakopoulos et al., 2018). This may increase the power of our method if more omics information are integrated. In addition, the cancer data used were from all patients of one type. However, there are different subtypes for multiple cancers. For example, BRCA is typically classified as luminal A, luminal B, triple-negative/basal-like, HER2-enriched, and normal-like subtypes, and HNSC contains HPV+ (human papillomavirus positive) and HPV– (human papillomavirus negative) subtypes (Vokes et al., 2015). Moreover, the characteristics, molecular profiles, or specific mutations are usually distinguished among different subtypes. Therefore, future work in dividing the cancer types into different subtypes to research the driver gene separately will be valuable.

In conclusion, Driver_IRW is easy to use for prioritizing cancer genes with the improved random walk–based method. We expect that our method will provide a valuable resource and can be amended in the future.

## Data Availability Statement

Publicly available datasets were analyzed in this study. This data can be found here: breast cancer (BRCA) with 1097 samples, head and neck squamous cell carcinoma (HNSC) with 522 samples, kidney renal cell cancer (KIRC) with 534 samples and thyroid cancer (THCA) with 513 samples from TCGA (https://portal.gdc.cancer.gov/).

## Author Contributions

P-JW conceived the algorithm, designed the method and drafted the manuscript, analyzed the data, and carried out the experiments. F-XW and C-HZ refined the idea, polished the English expression, and revised the paper. JX and YS participated in the design and revision of the research. JW participated the discussion and the coordination of the study. All authors read and approved the final manuscript.

## Conflict of Interest

The authors declare that the research was conducted in the absence of any commercial or financial relationships that could be construed as a potential conflict of interest.

## Abbreviations

TCGA: The Cancer Genome Atlas
ICGC: International Cancer Genome Consortium
SNVs: Single Nucleotide Variants
CNVs: Copy Number Variations
Indels: Insertions and Deletions
BMR: Background Mutation Rates
BRCA: Breast Cancer
HNSC: Head and Neck Squamous Cell Carcinoma
KIRC: Clear Cell Kidney Carcinoma
THCA: Thyroid Carcinoma
PCC: Pearson Correlation Coefficients
DC: Degree Centrality
BC: Betweenness Centrality
KC: Katz Centrality.
